# Exploring Clinicians’ and Patients’ Acceptance and Utilization of a Digital Solution to Support Individualized Care in Diabetes Specialist Outpatient Care (DigiDiaS): Qualitative Study

**DOI:** 10.2196/70301

**Published:** 2025-07-08

**Authors:** Maria Aadland Mollestad, Annesofie Lunde Jensen, Heidi Holmen, Tone Singstad, Eirik Årsand, Jacob Andreas Winther, Astrid Torbjørnsen

**Affiliations:** 1 Department of Nursing and Health Promotion, Faculty of Health Sciences, OsloMet—Oslo Metropolitan University, Postboks 4, St Olavs plass, Oslo, 0130, Norway, +4767235266; 2 SDCA—Steno Diabetes Center Aarhus, Aarhus University Hospital, Aarhus, Denmark; 3 Department of Clinical Medicine, Aarhus University, Aarhus, Denmark; 4 The Intervention Centre, Oslo University Hospital, Oslo, Norway; 5 Division of Medicine, Akershus University Hospital, Lillestrøm, Norway; 6 Department of Computer Science, Faculty of Science and Technology, UiT The Arctic University of Norway, Tromsø, Norway

**Keywords:** telemedicine, eHealth, mHealth, patient-reported outcome measures, outpatient care, patient-centered care, diabetes mellitus, type 1 diabetes, interpretive description

## Abstract

**Background:**

With the increasing prevalence of type 1 diabetes alongside limited health care resources, the need for more sustainable health care services is apparent. Central to ensuring the standard of diabetes care while simultaneously optimizing resource utilization is improved patient-clinician communication and the provision of individualized care. Digital outpatient solutions incorporating patient-reported outcome measures (PROMs) have been introduced in diabetes outpatient care over recent years; however, features and delivery methods differ, and existing studies on their use and perceived clinical value are limited. Furthermore, clinicians’ acceptance has been suggested as a key factor in the sustainability of digital solutions. Thus, to support the implementation of digital outpatient solutions perceived as valuable by clinicians and patients, we need more knowledge about how they are accepted and utilized in clinical practice.

**Objective:**

This study investigates how clinicians and patients with type 1 diabetes accept and utilize a digital outpatient solution to support individualized care in the context of full-scale implementation at a diabetes specialist outpatient clinic. Furthermore, we aim to explore the synchronous interaction between patients and clinicians when they are allowed to prepare through the filling and reviewing of asynchronous PROMs before consultations.

**Methods:**

This qualitative study uses interpretive description as a methodological approach. The digital outpatient solution features various components, including PROM questionnaires, asynchronous chat, remote consultations, e-learning, and information distribution. Data were collected through semistructured interviews with 10 clinicians and 20 patients with type 1 diabetes and observations of consultations. The data from the patient and clinician interviews (267 A4 pages) were analyzed separately before being jointly analyzed in the context of the findings from the observations (40 A4 pages).

**Results:**

Our analysis resulted in the following three main themes that describe the interplay between clinicians’ and patients’ acceptance, utilization, and perceived clinical value of a digital outpatient solution: (1) clinicians’ acceptance of the digital outpatient solution influences patients’ acceptance, (2) variations in the use of different features influence the extent of individualized care, and (3) clinicians’ and patients’ utilization influences perceived care efficiency and quality. Those who demonstrated higher acceptance and more extensive utilization reported that the solution was more valuable in enhancing individualized care efficiency and quality.

**Conclusions:**

This study highlights the interplay between clinicians’ and patients’ acceptance, utilization, and perceived clinical value of a digital outpatient solution in diabetes specialist outpatient care. Our findings suggest that when clinicians and patients understand why and how digital solutions are used, such solutions can enhance care efficiency and quality, contributing to sustainable health care. Future research should aim to gain an in-depth understanding of clinicians’ and patients’ acceptance, as well as the effectiveness of change management strategies when implementing digital outpatient solutions in diabetes specialist outpatient care.

## Introduction

### Background

As of 2022, about 8.75 million individuals worldwide were living with type 1 diabetes [1], a number predicted to increase to between 13.5 and 17.4 million by 2040 [2]. The rising global prevalence of type 1 diabetes, combined with limited health care resources, poses significant challenges to health care systems worldwide in their effort to provide high-quality care [3]. This escalating pressure calls for more sustainable health care services to ensure high diabetes care standards while simultaneously optimizing resource utilization. Thus, facilitating efficient communication between patients and their health care providers, emphasizing patients’ individual needs, and providing timely support will be increasingly important in the immediate future. Emerging digital solutions designed to promote patient communication and evaluate health status to individualize care offer opportunities and potential strategies to navigate this successfully [3,4].

An important aspect of diabetes care involves educating and supporting patients in developing sufficient self-management skills [5]. Diabetes self-management, which includes maintaining blood glucose levels within the target range, adopting a healthy lifestyle, and managing potential psychological issues, can help minimize the risk of complications and improve quality of life [6,7]. However, patients’ required self-management support level varies significantly, underlining the need for person-centered and individualized care [5]. Patient involvement becomes increasingly important, and digital solutions can be tailored to enable patients to participate actively in their treatment and self-management. One way of involving patients is through digital patient-reported outcome measures (PROMs), which entail collecting health information directly from patients without interpretation by a health care professional [8]. Relevant PROMs in type 1 diabetes may encompass patients’ perceived glycemic control, self-management activities, and diabetes distress. PROMs can provide a comprehensive understanding of patients’ needs and provide valuable information for clinicians when delivering person-centered and individualized care [9,10]. Simultaneously, PROMs can foster a self-reflection process within patients, leading to increased engagement in their treatment and self-management [9].

In recent years, digital outpatient solutions with PROMs have been developed and examined in diabetes specialist outpatient care [11-13]. However, according to a scoping review, these solutions’ features and delivery methods vary [4]. Some digital PROM-based outpatient solutions that aim to individualize care seem to focus on the psychological aspects of living with type 1 diabetes [14,15], while others also include other factors, such as glucose monitoring, self-management activities, and lifestyle [13,16]. The delivery methods range from patients completing the PROMs at a touch screen at the clinic to utilizing web-based solutions [4]. Furthermore, the literature on how the solutions are used is limited, mainly comprising small pilot and feasibility studies [4], underscoring the need for further research.

### DigiDiaS—Individualizing Diabetes Care Through a Digital Solution

In November 2021, a multicomponent digital outpatient solution was implemented at the diabetes specialist outpatient clinic at Akershus University Hospital in eastern Norway through the DigiDiaS project [17]. The solution mainly relies on PROMs entailing glucose monitoring, diabetes distress, and self-management activities. However, it also incorporates additional features aimed at individualizing care and supporting communication between clinicians and patients with type 1 diabetes, such as asynchronous chat and video consultations. For patients, use of the solution is voluntary and requires their consent upon app download. As of April 2024, 1354 out of approximately 1800 patients with type 1 diabetes had been included in the digital outpatient solution. All clinicians at the outpatient clinic utilize the digital outpatient solution, comprising diabetes specialist nurses and physicians working with type 1 diabetes patients.

For digital solutions aiming to individualize care to be implemented successfully, they must be accepted and utilized by both clinicians and patients, and clinicians’ acceptance has been suggested as a key factor in new digital solutions’ sustainability [18,19]. Acceptability, a core concept in digital health [20], can be defined as a multifaceted construct reflecting the extent to which people delivering or receiving a health care intervention consider it appropriate [19]. This acceptability can, in turn, influence utilization in the sense of user engagement and intervention effectiveness [20]. While previous research states that patients in diabetes outpatient care generally seem to welcome the use of digital PROMs [14,16,21,22] and the opportunity to self-report concerns [23,24], clinicians’ acceptance and perceptions of such measures’ usefulness vary [11,12,16]. Thus, we need more knowledge about how digital solutions are being accepted and utilized by clinicians and patients in diabetes specialist outpatient care [4]. Furthermore, no prior research has explored the synchronous interaction between clinicians and patients after asynchronous preparation using PROMs in a digital solution [4]. Consequently, the clinical value of using digital PROM-based solutions in diabetes care has yet to be established in terms of enhancing care quality and sustainable utilization of health care resources.

### Objective

This study investigates how clinicians and patients with type 1 diabetes accept and utilize a digital solution to support individualized care in the context of full-scale implementation at a diabetes specialist outpatient clinic. Furthermore, we aim to explore the synchronous interaction between patients and clinicians when they are allowed to prepare through the filling and reviewing of asynchronous PROMs before consultations.

## Methods

### Design

This qualitative study uses interpretive description as a methodological approach, comprising a framework developed to conduct research arising from clinical practice and aiming to improve practice [25]. The interpretive description process is inductive and pragmatic, providing flexibility to adapt the research process to the specific research questions [25]. Our study is embedded within a larger multimethod prospective observational study investigating digital PROMs in diabetes specialist outpatient care [17].

### The Multicomponent Digital Outpatient Solution

The previously detailed solution [17] was facilitated via Dignio Connected Care, offering web-based software used by clinicians and an app for patients [26], along with JOIN Norsk Helsenett, which provides a platform for video consultations [27]. Clinicians received training on the use of the digital outpatient solution, with ongoing support provided during and after the implementation process. Clinicians use the web-based Dignio platform to review completed PROM questionnaires from patients before a scheduled consultation, assign tasks, provide e-learning, and distribute digital information to individual patients and groups. During the outpatient clinic’s operating hours on weekdays, diabetes specialist nurses asynchronously respond to chat messages from patients, consulting a physician if necessary. On the patients’ side, the solution’s app enables them to complete assigned PROM questionnaires before consultations, receive information and e-learning, cancel consultations, reschedule or change upcoming consultations at the outpatient clinic to phone- or video consultations, and chat asynchronously with their clinicians as needed. The chat feature is mainly used by patients to report and ask questions about nonacute medical issues (self-initiated patient-reported outcomes) and submit practical queries. Should immediate assistance be required, patients are instructed to either call the clinic directly or dial the national emergency number.

**Figure 1.**
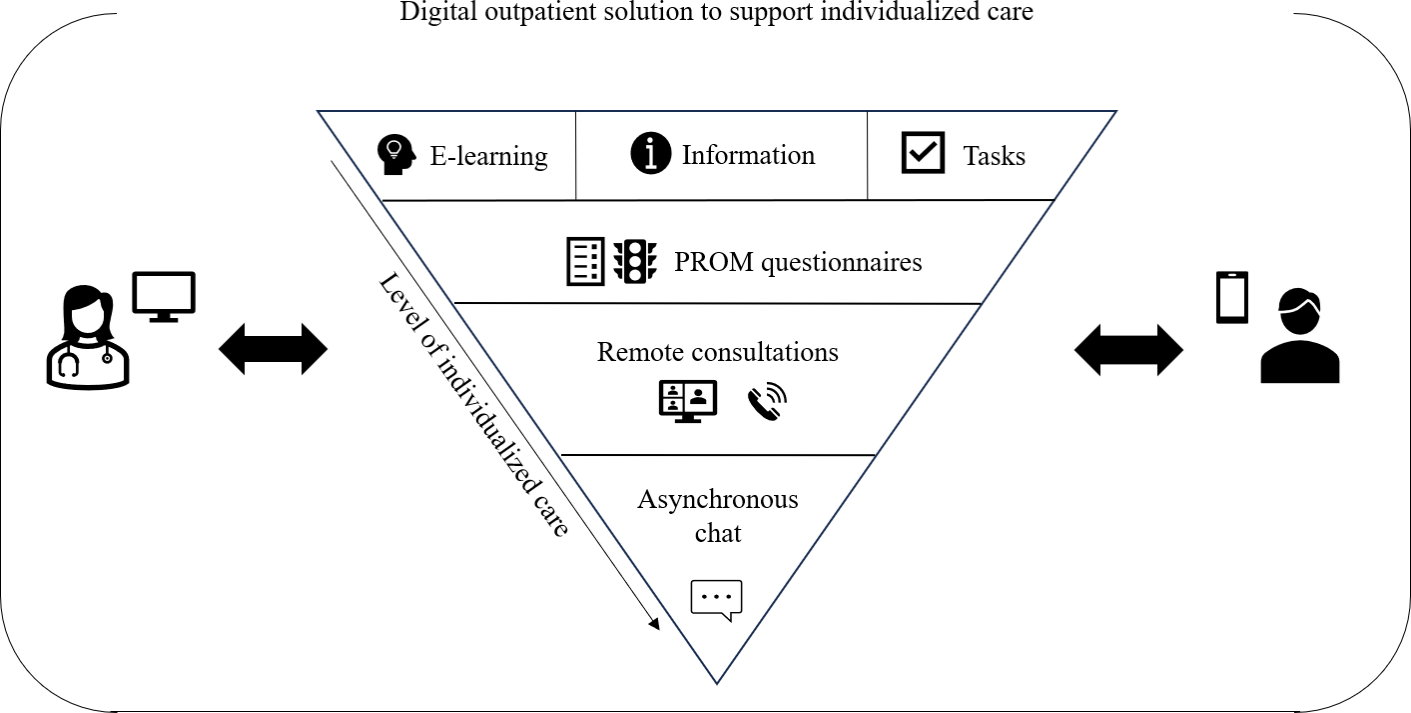
The digital outpatient solution aims to individualize care through improved patient-clinician communication. Each feature can be used based on an individual patient’s need for support. The triangular presentation illustrates the different levels of individualized care that each feature promotes, from uniform to highly individualized features. For example, while e-learning is a uniform feature received by those patients in need, the asynchronous chat is highly individualized and tailored to address a specific question at hand. PROM: patient-reported outcome measure.

**Figure 2.**
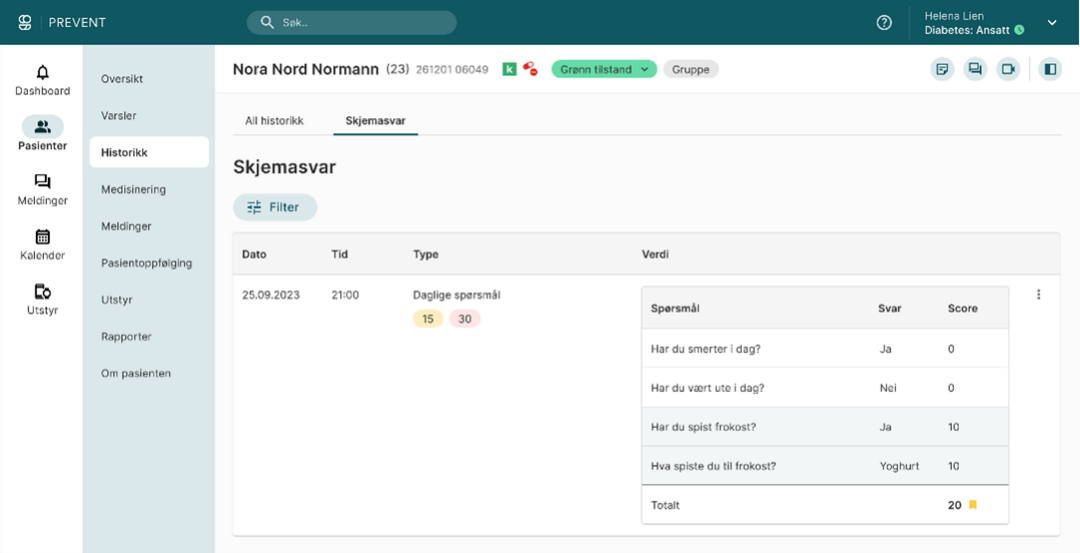
Screenshot from the dashboard used by clinicians. The screenshot is from a demo user and is intended as an example to illustrate the design.

**Figure 3.**
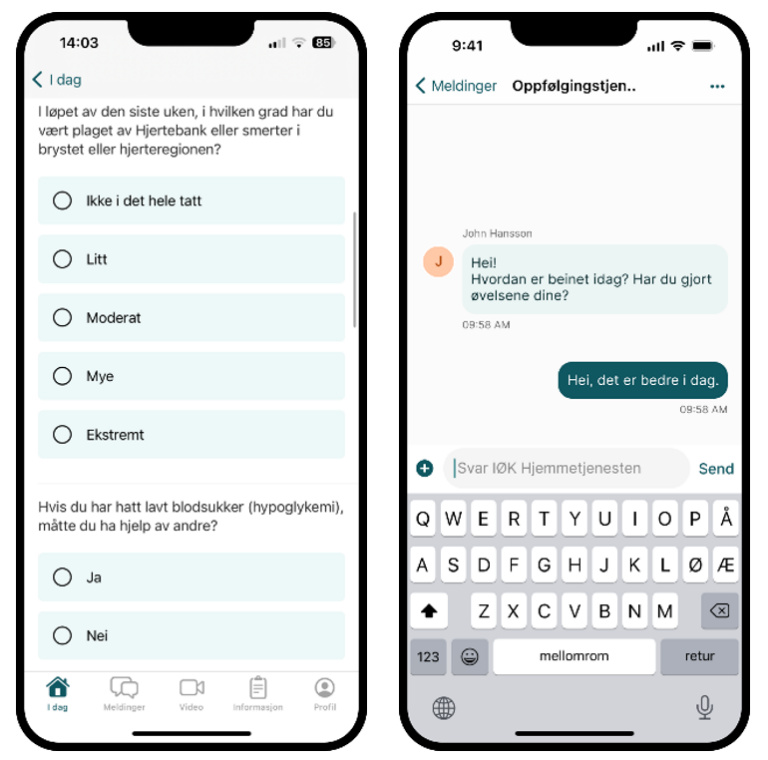
Screenshots from the app used by patients. The screenshot on the left illustrates how the patient-reported outcome measure questionnaire appears in the app, while the screenshot on the right shows an example of a chat conversation. These screenshots are from a demo user and are intended to illustrate the design.

The PROM questionnaire sent to patients before consultations is a modified and translated version of the DiabetesFlex Care PROM questionnaire originating from Denmark [13]. The questionnaire is multidimensional and is triaged based on preset thresholds using a traffic light system, which indicates patient responses’ severity [13]. Norwegian adjustments to the Danish version were made based on a user involvement study conducted at Akershus University Hospital, involving patients, diabetes specialist nurses, endocrinologists, management, and researchers [23]. In addition to PROMs, the questionnaire contains open text boxes for subjective responses. Figure 1 illustrates the digital outpatient solution’s different features. Figure 2 shows a screenshot of the web-based digital platform used by clinicians, while Figure 3 displays screenshots of the app used by patients.

### Participant Recruitment

Clinicians working with patients with type 1 diabetes at the outpatient clinic were recruited through convenience sampling [25], and the clinic manager invited them to participate. They were recruited based on availability, as we wanted to interfere as little as possible with clinical practice. All 10 clinicians invited to participate in the study, comprising 6 diabetes specialist nurses and 4 physicians, consented to participate. This represented approximately half the physicians and all the diabetes specialist nurses at the outpatient clinic, excluding the clinic manager and 1 newly hired nurse. Patients invited were adults with type 1 diabetes who had consented to use the digital outpatient solution and had completed the PROM questionnaire before their next consultation. Patients were recruited through purposive sampling [25] to ensure variation in gender, age, ethnicity, and frequency of digital interactions. Information about the study was sent to patients via the digital outpatient solution before their next upcoming consultation. At the start of the consultation, their clinician asked whether they wanted to participate. Of the 23 patients invited to participate, 20 consented. Written consent was obtained from all study participants.

### Data Collection

The first author collected data through observations and semistructured interviews between August 2023 and April 2024. An observation guide (Multimedia Appendix 1) and interview guides (MultimediaAppendices 2  3) were developed based on informal observations that the first author conducted at the outpatient clinic between February and March 2023, as well as aspects of the Method for Assessment of Telemedicine framework [28]. The guides were reviewed in a reference group comprising a user representative, researchers, clinicians, and management from the outpatient clinic. The observation guide facilitated insight into how the digital outpatient solution was used to individualize care, while the interviews sought to capture both clinicians’ and patients’ perspectives on their utilization of possibilities provided by the digital outpatient solution.

Scheduled consultations involving all participating patients and 6 out of 10 participating clinicians were observed. The observed consultations took place at the clinic (17), over the phone (2), or via video (1). Observations of remote consultations were conducted on-site, in the same room with the clinician at the outpatient clinic. Observation notes (40 A4 pages) were written down manually in the observation guide, digitally recorded, and stored. The notes in the observation guide contained descriptions of the type of consultation, topics discussed, context, use of PROM responses during the consultations, and analytical thoughts.

Individual semistructured interviews with clinicians were conducted before the observations, whereas patient interviews were conducted within 2 weeks after the observed consultations. The interviews were conducted at the clinic, over the phone, or via video based on participants’ preferences regardless of whether they were clinicians or patients. During the interviews, participants were asked questions about their attitudes toward the use of health technology in general, experiences with the implementation of the digital outpatient solution and using different features, the solution’s perceived usefulness, and potential improvements. Clinician interviews lasted between 28 and 52 minutes (median 37 minutes), and patient interviews lasted for 20-47 minutes (median 28 minutes). All interviews were transcribed verbatim (267 A4 pages).

### Data Analysis

**Figure 4.**
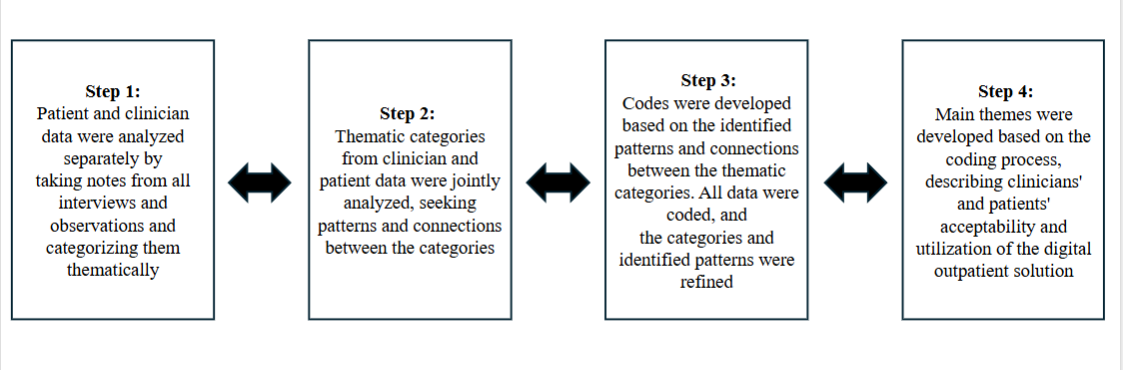
Illustration of the dynamic process of the constant comparative analysis.

Data analysis was conducted in line with interpretive description [25]. We concurrently collected data and conducted a constant comparative analysis. This made the process dynamic by moving back and forth between the different steps of the analysis, as illustrated in Figure 4.

The analysis aimed to inductively explore how clinicians and patients utilize a digital outpatient solution to individualize care. Through our interpretation process, we revealed a strong link between acceptability and utilization. In the final step of the analysis, 3 main themes were developed based on the coding process that describe clinicians’ and patients’ acceptance and utilization of the digital outpatient solution. The theoretical framework for acceptability was used to determine that the findings pertain to acceptance and utilization [19]. The first author conducted the analysis, with ALJ, HH, and AT contributing to analytical discussions during each step. These themes were discussed further with the remaining authors. NVivo 14 software (Lumivero) was used to manage and code the data [29], supplemented by visual exploration techniques to identify connections and patterns in the data. The visual exploration process involved positioning the thematic categories in relation to each other to identify patterns in clinicians’ and patients’ acceptance and utilization of the digital outpatient solution.

### Credibility

To ensure the credibility and epistemological integrity of our study, we have provided a comprehensive description of the study design and analysis process [25]. We aimed to establish representative credibility through prolonged engagement with the outpatient clinic prior to data collection. Furthermore, we enhanced the credibility of our findings by triangulating data, collecting information from multiple sources [30]. By thoroughly describing our results, we aim to demonstrate our analytic logic and interpretive authority to ensure trustworthiness [25].

### Ethical Considerations

The study was assessed by the Norwegian Agency for Shared Services in Education and Research (reference no. 456954) and the data protection officer at Akershus University Hospital (22/09709‐13). The project did not require approval by the regional committees for medical and health research ethics (reference no. 407539).

We used a service for sensitive data (TSD), developed at the University of Oslo, which was designed for storing and processing sensitive data in compliance with the Norwegian Personal Data Act and Health Research Act [31]. Written consent was obtained either on paper and stored securely, or digitally via Nettskjema [32], requiring a governmental ID portal login to access the information, and stored in TSD. All interviews were audiorecorded using the Nettskjema-diktafon app [33], a solution that immediately transfers all audio files encrypted for storage in TSD. MAM transcribed the interviews using the f4transkript program [34]. AT reviewed all recordings and transcripts throughout the analytical process, informing the interpretations. The privacy and confidentiality of research participants’ data and identities were maintained.

## Results

### Participants’ Characteristics

The patients’ characteristics, including their use of the digital outpatient solution, are shown in Table 1, and the clinicians’ characteristics, including their diabetes care experience, are shown in Table 2.

**Table 1.** Characteristics of patients and their use of diabetes technology and the digital outpatient solution (N=20).

Characteristics and usage	Values
Gender (female), n (%)	10 (50)
Age (years), median (minimum-maximum)	36 (18‐65)
Diabetes duration (years), median (minimum-maximum)	21 (1‐50)
Glucose monitoring, n (%)	
Continuous glucose monitor	19 (95)
Glucometer	1 (5)
Treatment, n (%)	
Insulin pump	12 (60)
Insulin pen	8 (40)
Number of completed PROM^a^ questionnaires, median (minimum-maximum)	1 (1‐7)
Number of chat conversations, median (minimum-maximum)	2 (0‐11)
Has used remote consultations, n (%)	12 (60)

a PROM: patient-reported outcome measure.

**Table 2.** Characteristics of clinicians and their diabetes care experience (N=10).

Characteristics and diabetes care experience	Values
Gender (female), n (%)	9 (90)
Diabetes specialist nurse, n (%)	6 (60)
Physician, n (%)	4 (40)
Nurses’ diabetes care experience (years), minimum-maximum	5‐25
Physicians’ diabetes care experience (years), minimum-maximum	5‐15

### Overview of the Identified Patterns

**Figure 5.**
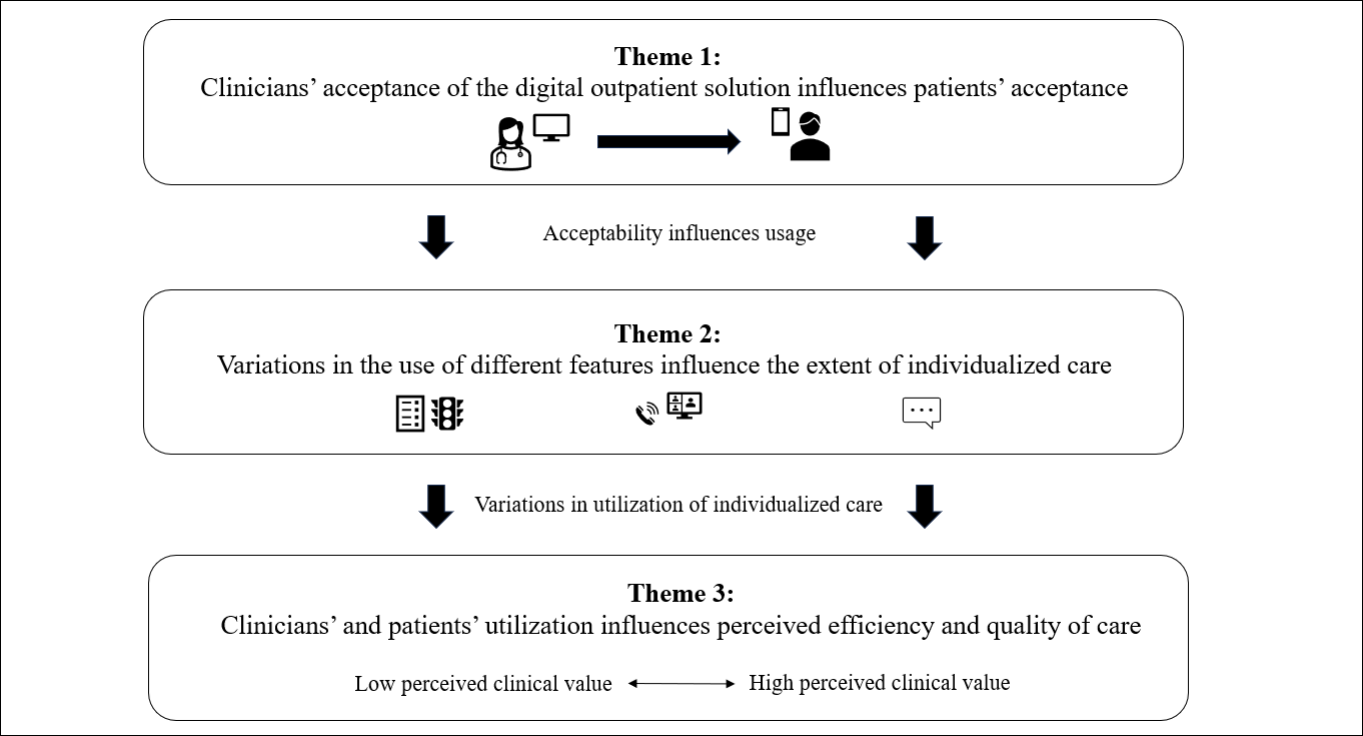
Illustration of how clinicians and patients accept and use the digital outpatient solution to support individualized care.

Our analysis identified three main themes that comprise a pattern describing the complex dynamics of how clinicians and patients accept and use the digital outpatient solution to support communication and individualize care: (1) clinicians’ acceptance of the digital outpatient solution influences patients’ acceptance, (2) variations in the use of different features influence the extent of individualized care, and (3) clinicians’ and patients’ utilization influences perceived care efficiency and quality. Figure 5 provides an overview of how the 3 themes are connected. Notably, the participants did not emphasize the possibility of assigning and receiving tasks, information, and e-learning, so it was not a focus of the analysis.

### Clinicians’ Acceptance of the Digital Outpatient Solution Influences Patients’ Acceptance

This theme describes how a variation was observed in how clinicians and patients accepted the digital outpatient solution, with an interplay between the two. Specifically, clinicians’ acceptance seemed to shape patients’ understanding of the solution’s use and, subsequently, their acceptance.

Clinicians’ understanding of the digital outpatient solution’s potential and their confidence in using its different features varied. They also expressed variations in their personal commitment toward mastering the digital outpatient solution’s features and adjusting their existing workflows. Some clinicians felt confident using the PROM questionnaires, chat, and remote consultations, viewing them as positive means to support individualized care:

*I really like it. Both the digital outpatient solution and I absolutely love video consultations because they shorten the time spent during consultations and we stay more on topic. And with the apps we use, we have access to much more data than before*.

[Clinician 1]

Conversely, other clinicians expressed uncertainty about using some features of the solution. While most felt comfortable using the chat and remote consultations, some were uncertain about how to use the PROM questionnaires before and during consultations, questioning whether the benefits outweighed the additional preparation time required of them and their patients:

*I feel it is a very comprehensive and somewhat time-consuming questionnaire for patients to fill out. I’m a bit worried that patients might find it to be too much work*.

[Clinician 10]

Furthermore, clinicians’ own understanding and acceptance of the solution seemed to affect how they introduced the solution to patients. Clinicians who indicated a solid understanding of the solution’s possibilities to individualize care expressed that they spent time explaining the various features to patients, during which they emphasized how it could enhance patients’ self-management awareness and improve communication with their clinicians. Patients who felt well informed about the solution’s potential also indicated higher acceptance levels:

*As we talked, he realized that this was his tool, and he could expect that I had read his responses before consultations. So, it suited him very well. He also understood that it was a way to make him more aware of his disease… Of course, it creates an expectation that I, as a clinician, should keep myself updated on their responses, but with him, I also experienced a positive expectation, that now we are talking, we can clear big and small problems out of the way early*.

[Clinician 3]

However, clinicians expressing less familiarity with the solution’s possibilities often focused on the chat feature as a means for patients to communicate with the clinic between consultations. Some reported that they did not mention the use of PROM questionnaires at all when presenting the solution to patients. This led to uncertainty among patients who received these questionnaires before consultations without adequate information about their purpose:

*It was a bit like, what is this, I thought…and it seemed that you had the option to decline to fill out the questionnaire. I believe there was an option to not fill out the questionnaire without losing the appointment. But I thought, OK, I’ll just fill out the form, it’s probably a questionnaire they have. But I thought the information was poor*.

[Patient 14]

Simply receiving the questionnaire without any clear instructions about its intended use was insufficient to gain patients’ acceptance. Clinicians’ and patients’ acceptance of the digital outpatient solution influenced further to what extent they used the different features to improve communication and individualized care.

### Variations in the Use of Different Features Influence the Extent of Individualized Care

This theme describes how clinicians and patients used PROM questionnaires, the option for remote consultation, and the chat feature to individualize care. It outlines the variations among participants’ use, as well as the interplay between clinicians’ and patients’ usage patterns.

#### PROM Questionnaires Can Facilitate Individualized Consultations Through Mapping Needs and Reflection

The most pronounced differences in use of the solution were related to use of patients’ PROM responses and how these responses influenced consultations. While some clinicians admitted to forgetting to review the patients’ PROM responses before a consultation occasionally, most clinicians made it a routine part of their consultation preparation. Some clinicians used PROM responses to tailor consultations and set agendas, ensuring that the patient’s needs were emphasized. When PROM responses were used actively, patients reported feeling more engaged in their treatment, with increased opportunities to discuss their concerns and priorities. This also included discussing positive PROM responses, as this could serve as a motivational factor in patients’ self-management process:

*I have previously felt that these consultations are a bit “so-so”. We talked about blood glucose monitoring and maybe not so much more. So, you don’t really know, and maybe the clinician doesn’t know either, which direction the conversation will take. So, the fact that I have already thought about what I want to discuss, and they are aware of my thoughts and feelings, has added great value to my consultations*.

[Patient 8]

When patients reported challenging issues through the questionnaire, clinicians could address these concerns specifically, improving their communication with their patients. However, some clinicians used the PROM responses less often during the consultations, which led their patients to question the value and purpose of the information they provided through the questionnaire:

*What then is the value of the questionnaire? It was not mentioned and none of the elements I reported were brought up... so I question the value of it. Questionnaires for the sake of questionnaires?*

[Patient 6]

If clinicians did not address patients’ PROM responses during the consultations explicitly, we observed that most patients did not bring it up. However, patients noted that this lack of use made it more challenging to engage actively in their consultations. Still, most patients used the PROM questionnaire as a tool for reflection to some extent to inform and guide their self-management process and prepare for consultations.

#### Consultations Tailored to Needs and Personal Preferences

With the digital outpatient solution’s implementation, most patients experienced a notable shift toward more individualized consultations. In addition to the use of PROM questionnaires, which affected the consultations’ content, the shift was facilitated by patients being asked whether they wanted to meet remotely with their clinicians by either phone or video. Both clinicians and patients agreed that certain matters, such as physical checks or discussing difficult or complex issues such as mental health challenges, were best addressed in person. However, many found remote consultations more convenient for simpler requirements, such as support when starting to use a new insulin pump:

*I have used it in the follow-up when patients have started using an insulin pump. Initially, a physical meeting is required to get started with the pump, but the subsequent follow-up is conducted digitally. This is possible because patients have uploaded their pump data. This allows us to see all the necessary information while they are at home, enabling us to make necessary adjustments*.

[Clinician 1]

However, both clinicians and patients expressed personal preferences regarding using remote consultations. While some preferred communicating remotely and used it regularly, others had a strong preference for in-person consultations:

*I have always chosen to decline that option because I prefer to speak with people face-to-face. I have used video meetings a lot for work and other settings, and that’s not a problem, but I feel much more comfortable face-to-face*.

[Patient 10]

Patients regularly chose between physical attendance at the clinic and video or phone consultations based on needs or personal preferences.

#### Chat Messages Facilitate Individualized Care When Needed

Most patients used the chat feature, which all the diabetes specialist nurses used consistently, facilitating targeted care within a short time frame. Chat was highlighted as the most frequently used feature of the digital outpatient solution. For clinicians, chat presented an opportunity to maintain contact with patients and even reduce consultation frequency if adequate follow-up could be conducted via chat:

*We reach more patients when they have the opportunity to send a message. We dare, in a way, to schedule their next consultation in half a year because they can write to us in the meantime if they need to*.

[Clinician 1]

When confronted with complex patient inquiries via chat that warranted comprehensive written responses or extensive dialogue with the patient, clinicians found transitioning to immediate remote consultations useful in providing the necessary support. These chat-initiated remote consultations, serving as an extension of the chat interaction, enriched the overall communication process:

*When the patients write messages, we see how extensive the issue is, whether it’s a short question, or if it’s a question that requires having a conversation around what the patient is wondering about. So, I often ask if we can have a conversation instead of answering a dissertation...And there is a lot of good feedback on that. I also think we can resolve issues quickly so that they don’t have to wait until their next scheduled consultation to get answers. So, I believe that we are promoting health more in the way we are working now*.

[Clinician 3]

The patients found it easier to contact the clinic through chat. Moreover, patients reported that chat supported their self-management, as they used it to address urgent concerns immediately instead of waiting for their next consultation or calling the clinic. Many patients viewed phone calls as a barrier, and they experienced a lower threshold for writing their questions in chat. Patients and clinicians used the chat feature for a range of queries, from addressing practical questions about upcoming consultations to discussing treatment-related matters, such as insulin adjustment. Chat also provided a sense of security for patients, knowing that help was just a message away:

*It certainly helps me to have the reassurance that I can simply send a message instead of having to call and wait for a response and all that, so it’s very convenient. Let us say I’m at work and wondering about something, I can just send a message, and I know I’ll get a response soon. Especially when I don’t have the opportunity to be on the phone, it’s a great comfort knowing there’s someone there to help*.

[Patient 4]

Although some patients had used chat only once or twice, or not at all, they reported a sense of security from knowing that they had the option to send a chat message when necessary. This underscores the chat feature’s latent value, demonstrating its significance even when not used actively.

### Clinicians’ and Patients’ Utilization Influences Perceived Care Efficiency and Quality

Both clinicians and patients acknowledged the digital outpatient solution’s potential clinical value in enhancing efficiency by streamlining communication and improving care quality through individualized follow-up based on patients’ needs. However, the extent of the perceived clinical value varied among clinicians and patients and was reflected in how they utilized different features of the solution. Clinicians’ and patients’ utilization, that is, their practical and effective use of the digital outpatient solution, appeared to influence their experiences regarding the solution’s benefits, with those demonstrating extensive utilization typically perceiving the solution as more valuable. Furthermore, this theme describes clinicians’ and patients’ perspectives on how the different features can contribute to enhanced care efficiency and quality.

The most prominent differences in clinicians’ and patients’ perceived clinical value were related to the PROM questionnaires. When utilized, both clinicians and patients found that the questionnaires helped focus on patient-identified key areas for managing their life with type 1 diabetes, simultaneously making consultations more efficient by prioritizing topics. From the patients’ perspective, the PROM questionnaires were also viewed as the clinic’s acknowledgment of the complex nature of living with type 1 diabetes. This comprehensive focus on all aspects of living with diabetes was believed to enhance care quality by enabling the patients to address challenging issues more comfortably:

*It’s easier to sit and write it on the phone than to bring it up in a consultation because the questionnaire is a lot about your thoughts and feelings, and it is not always easy to talk about… and when you are in a consultation, they often ask you how you are doing and then you usually just say that it’s going well even though it’s not*.

[Patient 11]

However, patients stressed that the extent to which clinicians utilized their responses was crucial in perceiving the questionnaire as valuable from the patients’ perspective. Although not all patients experienced remote consultations, most recognized their clinical value in increasing efficiency. Some patients also expressed a wish to try remote consultations in the future, particularly if circumstances prevented them from meeting clinicians in person at the outpatient clinic. Both patients and clinicians appreciated remote consultation’s potential to increase efficiency and flexibility. Patients clearly benefited from a reduction in travel time, while some clinicians valued the possibility of working remotely. Both parties also noted that remote consultations tended to be more concise, without the usual small talk evident during in-person consultations. However, this led to a few clinicians expressing concerns about potentially missing important information, with some worrying that care quality provided remotely was not as high as in person. Most patients did not share the same concern, as they would choose clinic consultations for issues they felt were better discussed in person.

Both clinicians and patients emphasized the chat feature’s role in elevating care efficiency and quality. Clinicians found that chat reduced the volume of calls to the clinic, allowing them to respond at their convenience, rather than being interrupted by calls during other tasks. Although responses from the clinic could take a few hours or even a couple of days, patients perceived the overall process as more efficient and flexible than calling. Furthermore, chat enabled clinicians to give more thought-out advice, differing from the immediate responses provided over the phone and enhancing the perceived quality of the responses provided to patients:

*After nearly two years of using the solution, we appreciate receiving written questions, as it allows us to respond at our convenience. This also allows us to thoroughly consider our response and consult with a colleague before replying if necessary. I think this is a good approach*.

[Clinician 4]

Patients appreciated the chat feature’s value in offering an accessible way to receive reassuring responses to their questions, thereby easing everyday management of their condition. Both clinicians and patients viewed the chat feature, largely driven by patient-initiated conversations, as a key element of the digital outpatient solution for individualizing care to meet patients’ needs.

## Discussion

### Principal Findings

This qualitative study provides insight into how clinicians and patients accept and utilize a digital outpatient solution in a diabetes outpatient clinic differently, highlighting an interplay between their usage patterns. Specifically, clinicians’ acceptance seems to shape patients’ understanding and acceptance of the solution’s resources, subsequently influencing clinicians’ and patients’ use. Furthermore, this seems to influence clinicians’ and patients’ perceived clinical value of the solution’s different features. Those demonstrating higher acceptance and more extensive utilization perceived the solution as more valuable, recognizing its potential to enhance care efficiency and quality.

To our knowledge, no extant literature has highlighted the interplay between clinicians’ and patients’ acceptance, utilization, and perceived clinical value of digital outpatient solutions during full-scale implementation in diabetes specialist outpatient care. The literature mostly encompasses pilot and feasibility studies, with an emphasis either on the patients’ or the clinicians’ perspective [4]. Although the literature in this field is limited, studies have indicated that patients generally embrace PROM-based digital outpatient solutions [16,22], whereas clinicians often express ambivalence and challenges related to time, resources, and care quality [11,12,16]. Our findings particularly align with how clinicians express ambivalence about using digital outpatient solutions, revealing variations in satisfaction and use of the solution. Moreover, our study goes further than extant studies in exploring the complex interplay between clinicians and patients, specifically by demonstrating that clinicians’ acceptance influences patients’ acceptance and their collective utilization of the solution. This interplay underscores the important role of clinicians’ understanding and acceptance of the digital outpatient solution for successful implementation [19].

The importance of clinicians’ acceptance has been emphasized in literature examining telehealth services in different contexts [18]. Regarding this specific multicomponent digital solution, our findings suggest that both clinicians and patients perceived combining different features to improve the efficiency and quality of individualized care as clinically valuable and appropriate for implementation in clinical practice [19]. However, variations in acceptability across individuals seemingly affect clinicians’ and patients’ perceived clinical value of each feature, influencing their utilization in the sense of user engagement and intervention effectiveness [20]. For instance, most participants used the chat feature and found it valuable for individualizing care, aligning with extant literature arguing that a chat feature can strengthen clinician-patient relationships in type 1 diabetes care [35]. Remote consultations were used based on individual preferences and needs and were valued particularly for their role in enhancing communication efficiency, a benefit also reported in earlier literature [36]. However, we observed wide variations in use and perceived clinical value of PROM questionnaires. Extant literature has argued that PROMs’ usefulness in clinical practice depends on patients reporting their concerns, clinicians’ proficiency in utilizing information, and both patients and clinicians perceiving them as useful [37]. This suggests that the effectiveness of using PROMs to individualize care depends on clinicians’ and patients’ ambition and capacity to leverage opportunities that PROMs offer. Without active engagement from both clinicians and patients, PROM questionnaires can be perceived as a burden rather than an asset, adding to workloads without enhancing perceived clinical value.

### Implications for Clinical Practice

#### Overview

To ensure successful implementation, it is crucial to address both human factors and organizational change [38]. We argue that an in-depth understanding of human factors, such as users’ acceptance, is essential when implementing a digital outpatient solution in diabetes care. Based on our findings, we emphasize the importance of clinic management in addressing the challenges and implications that clinicians and patients encounter during implementation, as these factors can significantly impact the solution’s sustainable utilization.

#### Importance of User Acceptance and the Clinicians’ Role in Implementation

Based on our findings, which underscore the important role of clinicians’ acceptance in the sustainable utilization of the digital outpatient solution, we argue that clinicians should be a prioritized group during implementation. When clinicians accept and understand how the digital outpatient solution can be utilized, they are in a better position to provide the necessary information for patients to start using it. Extant literature has recognized clinicians’ role in implementing digital outpatient solutions successfully [18] and has suggested further exploration of workflow changes that clinicians encounter when implementing digital solutions in diabetes care [4]. Furthermore, clinicians’ role has also been highlighted in the context of other diagnostic groups, such as epilepsy, in which it has been argued that PROM-based follow-up represents substantially changed outpatient care and that implementation success is highly dependent on clinicians’ daily management of the solution [38].

#### The Need for Organizational Involvement and Strategy

Ensuring clinicians’ acceptance and competence to provide the necessary information and education to patients about how to utilize digital outpatient solutions seems to require organizational strategies that emphasize education, training, and ongoing support throughout and after implementation. As part of the implementation of this digital outpatient solution, all clinicians underwent training and were offered ongoing support as needed. Nevertheless, the persistence of some clinicians’ hesitance to change implies that more comprehensive organizational involvement may be necessary for optimal implementation. Strategies that address clinicians’ individual support needs might help foster more universal acceptance among clinicians.

Furthermore, our findings indicate that the extent of organizational involvement required for implementation to be successful can differ, depending on the effort required from clinicians and patients to adapt to the new practices. In this study, the implementation of the chat feature and offering patients the choice of remote consultations were viewed as particularly valuable. However, the implementation of PROM questionnaires required considerable changes in clinicians’ workflow and their care delivery methods, while concurrently requiring increased patient involvement. Extant literature has highlighted the need for clinicians to adapt their care methods when implementing PROMs in diabetes care [12] and that the efficiency of using PROMs in clinical practice seems to increase in line with clinicians’ and patients’ understanding of why and how they are used [37]. Consequently, we suggest that more structured education and follow-up from clinic management are needed on the use of PROM questionnaires, compared with other, more easy-to-use features of the digital outpatient solution, such as the chat feature and remote consultations.

Change management, a systematic approach to managing the human elements of change [39], could inspire strategies to ensure utilization when implementing a digital outpatient solution. This approach involves a set of processes designed to guide an organization through a transition [39] and has been applied in other contexts implementing digital services [40], as well as other settings in health care services [41]. We propose that using a change management approach adapted to the specific context could facilitate future implementations of digital outpatient solutions in diabetes specialist outpatient care. However, implementing change notably often requires more time and resources than anticipated, particularly under pressure [42]. Given the increasing patient population in diabetes care [2], as well as the constraints of limited health care resources, potential implementation challenges should be considered before introducing new digital outpatient solutions in diabetes specialist outpatient care. Thoughtful consideration of potential implementation challenges and proactive planning for their resolution could enhance the likelihood of successful implementation.

### Limitations

Despite providing valuable insights, this study has some limitations. The data, collected from 1 single diabetes outpatient clinic and specific to 1 particular digital outpatient solution, may not present patterns transferable to other settings or digital solutions. However, we did find that patients and clinicians were reporting similar experiences in other studies, although the content and delivery methods in their digital outpatient solutions differed [11,12,16,22]. These studies were also conducted in Scandinavian countries, providing a patient population and health care setting comparable with our study.

Another potential limitation is that some of the clinicians interviewed in this study also participated in the user involvement study previously conducted at Akershus University Hospital [23], which aimed to develop this digital outpatient solution and the care trajectory. The clinicians’ involvement in the solution’s development process may have influenced their attitudes and understanding of its use. This implies that not all clinicians may have had the same foundation or knowledge to utilize the solution, potentially contributing to observed variations in acceptance, utilization, and perceived clinical value. Still, this variation in clinicians contributes to bringing different perspectives with the digital solution into the study, and the findings emphasize the importance of understanding why and how such solutions are used for their effective utilization.

All patients recruited for this study had used the solution to some extent, having completed at least 1 PROM questionnaire. We chose to focus on this group to gain insight into the utilization of the different features. However, we acknowledge a limitation in that we have not considered patients who have accepted using the digital outpatient solution but have not done so for various reasons. Understanding the barriers that these patients face in using the solution could provide valuable insight, potentially helping to facilitate a smoother transition to digital follow-up for patients currently not using the solution.

Finally, as this is a qualitative study, we are gaining deep insight into how the solution is utilized in clinical practice. Although our sample size concerning the clinicians could have been larger, it still contained 96 A4 pages of transcribed data. This shows a high degree of information power [43]. However, using a mixed methods design or further investigating patients’ and clinicians’ acceptability and utilization quantitatively could deepen our understanding and enhance the validity and reliability of our findings.

### Conclusions

This study explored the interplay between clinicians’ and patients’ acceptance, utilization, and perceived clinical value of a digital outpatient solution in diabetes specialist outpatient care. We identified variations in the use of the solution’s features, suggesting that certain features might require greater knowledge and efforts from clinicians and patients to be utilized efficiently, particularly the use of PROM questionnaires. Our findings imply that when clinicians and patients understand why and how such solutions are used, they can enhance care efficiency and quality, contributing to sustainable health care services. We argue that an in-depth understanding of clinicians’ acceptance is essential when implementing a digital outpatient solution. Furthermore, using a change management approach, adapted to the specific context, could facilitate future implementations to ensure sustainable utilization. Future research should look deeper into clinicians’ and patients’ acceptance, as well as use of change management strategies when implementing digital outpatient solutions in diabetes specialist outpatient care.

## Supplementary material

10.2196/70301 Multimedia Appendix 1 Observation guide.

10.2196/70301 Multimedia Appendix 2 Interview guide—clinicians.

10.2196/70301 Multimedia Appendix 3 Interview guide—patients.

## Abbreviations

PROM: patient-reported outcome measure
