# Supplementary material for: Exploring Clinicians’ and Patients’ Acceptance and Utilization of a Digital Solution to Support Individualized Care in Diabetes Specialist Outpatient Care (DigiDiaS): Qualitative Study
Source: JMIR Hum Factors. 2025 Jul 8;12:e70301. doi: 10.2196/70301 (PMC12262153; doi:10.2196/70301)
Supplement: Multimedia Appendix 1 [file humanfactors-v12-e70301-s001.docx]

**Appendix 1. Observation guide**

Participant no., patient: Date and time used:

Participant no., clinician: Location:

| **Description** | | **Reflections** |
| --- | --- | --- |
| Patient:  Clinician:  Surroundings: | | What have I learned, and did I learn something that I didn't know before?  Was there anything that surprised me or that I didn't understand?  Did something happen that I couldn't get hold of that I can be aware of next time? |
| Topics |  |  |
| Interaction |  |  |
| PROM communication |  |  |
| Patient and therapist behavior |  |  |
| Use of technology |  |  |
| Other |  |  |
